# Anti-V3 Monoclonal Antibodies Display Broad Neutralizing Activities against Multiple HIV-1 Subtypes

**DOI:** 10.1371/journal.pone.0010254

**Published:** 2010-04-21

**Authors:** Catarina E. Hioe, Terri Wrin, Michael S. Seaman, Xuesong Yu, Blake Wood, Steve Self, Constance Williams, Miroslaw K. Gorny, Susan Zolla-Pazner

**Affiliations:** 1 Department of Pathology, New York University Langone School of Medicine, New York, New York, United States of America; 2 Veterans Affairs New York Harbor Healthcare System, New York, New York, United States of America; 3 Monogram Biosciences, Inc., South San Francisco, California, United States of America; 4 Department of Medicine, Division of Viral Pathogenesis, Beth Israel Deaconess Medical Center, Harvard Medical School, Boston, Massachusetts, United States of America; 5 Public Health Sciences Division, Statistical Center for HIV/AIDS Research and Prevention, Fred Hutchinson Cancer Research Center, Seattle, Washington, United States of America; University of California San Francisco, United States of America

## Abstract

**Background:**

The V3 loop of the HIV-1 envelope (Env) glycoprotein gp120 was identified as the “principal neutralizing domain” of HIV-1, but has been considered too variable to serve as a neutralizing antibody (Ab) target. Structural and immunochemical data suggest, however, that V3 contains conserved elements which explain its role in binding to virus co-receptors despite its sequence variability. Despite this evidence of V3 conservation, the ability of anti-V3 Abs to neutralize a significant proportion of HIV-1 isolates from different subtypes (clades) has remained controversial.

**Methods:**

HIV-1 neutralization experiments were conducted in two independent laboratories to test human anti-V3 monoclonal Abs (mAbs) against pseudoviruses (psVs) expressing Envs of diverse HIV-1 subtypes from subjects with acute and chronic infections. Neutralization was defined by 50% inhibitory concentrations (IC_50_), and was statistically assessed based on the area under the neutralization titration curves (AUC).

**Results:**

Using AUC analyses, statistically significant neutralization was observed by ≥1 anti-V3 mAbs against 56/98 (57%) psVs expressing Envs of diverse subtypes, including subtypes A, AG, B, C and D. Even when the 10 Tier 1 psVs tested were excluded from the analysis, significant neutralization was detected by ≥1 anti-V3 mAbs against 46/88 (52%) psVs from diverse HIV-1 subtypes. Furthermore, 9/24 (37.5%) Tier 2 viruses from the clade B and C standard reference panels were neutralized by ≥1 anti-V3 mAbs. Each anti-V3 mAb tested was able to neutralize 28–42% of the psVs tested. By IC_50_ criteria, 40/98 (41%) psVs were neutralized by ≥1 anti-V3 mAbs.

**Conclusions:**

Using standard and new statistical methods of data analysis, 6/7 anti-V3 human mAbs displayed cross-clade neutralizing activity and revealed that a significant proportion of viruses can be neutralized by anti-V3 Abs. The new statistical method for analysis of neutralization data provides many advantages to previously used analyses.

## Introduction

Gp120, the surface subunit of the HIV-1 envelope (Env) glycoprotein, is a critical target for antibodies (Abs) that neutralize the virus and prevent infection (reviewed in [1]). Gp120 is bound non-covalently to the transmembrane subunit gp41, and the two glycoproteins are expressed on the virion surface as heterotrimers. Gp120 serves as the virus attachment protein by binding to CD4 and the chemokine receptors CCR5 or CXCR4. Because of these crucial functions in the virus infectious process, it is logical that gp120 is a desired target for neutralizing Abs. However, gp120 displays astonishing agility in evading Ab neutralization [2], [3], [4], [5], [6]. Indeed, HIV-1 gp120 is notorious for its genetic and antigenic variability, while at the same time, its conserved regions are poorly immunogenic and/or are not accessible on the surface of the virion at all times [5], [7], [8], [9], [10], [11], [12].

Conserved Ab epitopes on gp120 have been identified based on their recognition by broadly neutralizing human mAbs (reviewed in [13]). Not surprisingly, these epitopes are located in the Env regions critical for virus infectivity, which include the CD4-binding site and the chemokine-receptor binding site which encompasses the stem of the second variable (V2) loop, the third variable (V3) loop, and the bridging sheet [14], [15], [16], [17], [18], [19]. Recent analyses of serum Abs from HIV-1+ subjects further confirm the importance of the epitopes in these receptor binding sites as targets of broadly neutralizing Abs [20], [21], [22], [23].

The present study evaluates the breadth and potency of virus neutralization by mAbs specific for epitopes in the V3 loop. The V3 loop was identified in the late 1980s as the principle neutralizing domain of HIV-1 [24], but was considered an inappropriate target for vaccines because this region, as its name indicates, is characterized by extreme sequence variability. This concept was supported by early studies showing that anti-V3 Abs raised in peptide-immunized goats and in HIV-1-infected chimpanzees were “type-specific”, as they were restricted in their reactivity among a limited number of laboratory-adapted virus strains. [25], [26]. Other studies further showed that V3 epitopes were cryptic or masked in many HIV-1 clinical isolates due to occlusion by glycans, the V1/V2 loops, and possibly other unidentified elements [9], [27], [28], [29]. In contrast, however, several studies demonstrated that mAbs and polyclonal serum Abs against V3 can display significant degrees of cross-neutralization against viruses within a single subtype and among multiple subtypes [7], [17], [18], [27], [30], [31], [32]. Given the fact that V3 is a part of gp120 that interacts with the chemokine receptors [33], [34] and that it determines CCR5 or CXCR4 usage [35], [36], [37], [38], V3 must retain conserved structural elements despite its sequence variation, and it must be exposed, at least transiently, to enable virus binding to the chemokine receptors. These features are likely to account for the ability of many anti-V3 Abs to recognize and neutralize diverse HIV-1 isolates.

More recently, a variety of studies have demonstrated that V3 is a structurally conserved domain. Thus, crystallographic and NMR studies show conserved features of V3 when bound to several human anti-V3 mAbs [37], [39], [40], [41], [42]. These structural studies are consistent with the single structure available for V3 in the context of gp120 [43], and they provide an explanation for how anti-V3 Abs can tolerate sequence changes in their epitopes, react immunochemically with a variety of V3 peptides and Env proteins, and display cross-clade neutralizing activity against primary isolates and pseudoviruses (psVs) [17], [18], [23], [30], [32], [44]. Nevertheless, the proportion of diverse viruses which anti-V3 Abs can neutralize and their breadth and potency against viruses from the various HIV-1 subtypes (clades) and from patients at different stages of infection remain controversial.

To address this issue in a comprehensive manner, HIV-1 neutralization experiments were conducted in two independent labs to test seven anti-V3 mAbs which were selected because they had previously been shown to display potent and cross-clade neutralizing activity [17], [18], [19], [45]. These mAbs were tested against a panel of 98 pseudoviruses (psVs) expressing Envs of HIV-1 subtypes A, AG, B, C, and D from patients with acute and chronic HIV-1 infections. Positive neutralization was determined for each mAb/psV pair on the basis of the commonly used cut-off criterion, the 50% mAb inhibitory concentration (IC_50_). In addition, a new statistically-based criterion was used which takes into account (a) the area under the mAb titration curve (AUC), (b) the slope of the titration curve, (c) the background neutralization from irrelevant control mAbs, and (d) the background from a control psV expressing Env from the amphotropic murine leukemia virus (aMLV).

## Materials and Methods

### Ethics Statement

The study was approved by the IRB of New York University School of Medicine. All subjects gave written informed consent.

### Monoclonal antibodies

The seven human anti-V3 mAbs examined in this study were developed using previously described cellular techniques [17], [18], [19], [45], [46]. In brief, Epstein-Barr virus transformed peripheral blood mononuclear cells from HIV-1-infected subjects producing V3-specific Abs were fused with the heteromyeloma SHM-D33, and the resulting hybridomas were cloned to monoclonality. Except for mAb 447-52D (designated here as 447) which was selected using a V3_MN_ peptide, the anti-V3 mAbs were selected using murine leukemia virus gp70-based fusion proteins (FPs) containing V3 loops from viruses of subtypes A or B [19], [47]. These seven anti-V3 mAbs were selected from among >50 anti-V3 mAbs generated in our lab to represent the most potent and cross-reactive neutralizing anti-V3. Human mAbs specific for parvovirus B19 [48] or the anthrax protective antigen (PA) were used as negative controls. The anti-PA mAbs were produced by cellular methods from cells derived from two different volunteers who received an experimental vaccine consisting of recombinant PA antigen (Gorny et al., unpublished data). All mAbs were purified from culture supernatants by protein A or protein G chromatography. Table 1 summarizes the properties of the mAbs tested in this study. The 11 mAbs were sent to the laboratories where the functional assays were performed without any designation of their specificities.

**Table 1:** Characteristics of human mAbs used for this study.

mAbs	Specificity	Isotype	Subtype of the infecting virus	Country of origin	Reference
2191	V3	IgG1 λ	B	**USA**	[19]
2219	V3	IgG1 λ	B	**USA**	[19]
2557	V3	IgG1 λ	CRF02_AG	**Cameroon**	[18]
2558	V3	IgG1 λ	CRF02_AG	**Cameroon**	[18]
3074	V3	IgG1 λ	CRF02_AG	**Cameroon**	[18]
3869	V3	IgG1 λ	nd	**Cameroon**	[46]
447	V3	IgG3 λ	B	**USA**	[17], [45]
860	parvovirus	IgG1 λ	-	**USA**	[48]
1418	parvovirus	IgG1 κ	-	**USA**	[48]
3685	Anthrax, PA	IgG1 λ	-	**USA**	§
3706	Anthrax, PA	IgG1 λ	-	USA	§

nd – not determined.

PA – protective antigen of anthrax.

§– Gorny et al., unpublished data.

### Neutralization assay with U87 target cells

The PhenoSense™ HIV neutralization assay was performed by Monogram Biosciences, Inc. using the U87 target cell line expressing CD4, CCR5, and CXCR4, as previously described [32], [49]. The U87 cell line was generated by Dr. Nathaniel Landau (New York University School of Medicine, New York, NY). This single round replication assay was used to test 57 psVs expressing cloned Env gene populations extracted from viruses in patients' plasma. The psVs were first treated with 2- or 3-fold serial dilutions of mAbs starting from 50 µg/ml, and then incubated with the U87 cells. After 72 hr, the levels of virus infection were assessed by measuring luciferase activity. In this assay, the anti-parvovirus mAb 860-55D (designated here as 860) and an aMLV Env-expressing psV were used as negative controls, whereas psVs expressing cloned Envs of SF162, JR-CSF, and NL4.3 were tested as positive controls.

### Neutralization assay with TZM.bl target cells

Neutralizing activities of the anti-V3 mAbs against 41 psVs bearing single cloned Envs from neutralization-sensitive viruses (Tier 1), from clade B and clade C primary isolates from recent infections (Tier 2), and viruses from chronic infections were measured using the TZM.bl cell line as target cells, as previously described [50], [51], [52]. Similar to the U87 assay described above, 2-fold serial dilutions of mAbs were prepared starting from 50 µg/ml and used to treat psVs. The mAb/psV mixtures were then incubated with the TZM.bl target cells, and luciferase activity measured 48 hr later. MAbs specific for parvovirus B19 (1418 and 860) or anthrax protective antigen (3685 and 3706) were tested as negative controls for this set of experiments.

### Analyses of neutralization data

For each mAb/psV combination, a polynomial regression (quadratic) model was used to best fit the titration curve. From each fitted titration curve, the IC_50_ and area under the curve (AUC) values were estimated to quantify neutralization potency. The IC_50_ value denotes the mAb concentration that corresponds with 50% neutralization in each fitted titration curve. AUC, on the other hand, is defined as an integration of the fitted curve over a chosen concentration range divided by the concentration range. AUC can be interpreted as the average neutralization within the given concentration range, with 1 as the maximal value representing 100% neutralization across the entire concentration range specified.

To allow for comparison among different experiments and different assays, AUC values were calculated over a fixed range of mAb concentrations (0.39–50 µg/ml). For each mAb/psV combination, we tested the none-zero null hypothesis that AUC was less than or equal to a constant c using the Wald test. The constant c was calculated as the mean AUC plus 2 standard deviations from the negative controls (aMLV and/or irrelevant mAbs) achieved in each set of experiments. The c values for the U87 and TZM.bl experiments were 0.06 and 0.18, respectively. The one-sided Wald test that takes into account the AUC and its variance determined whether neutralizing activity of each test mAb/psV combination was statistically significant at the confidence level of 0.001. However, because the AUC values summarize the titration curves without assuming a sigmoidal curve shape, an additional criterion was used to ensure the detection of dose-dependent neutralization among the titration curves with relatively low AUC values (AUC≤ constant c+0.15), i.e., the presence of a positive slope between 30 and 40 µg/ml of mAb. All computations were done with free statistical software R (http://www.r-project.org/), except for the t test or the non-parametric one-way ANOVA test which were performed using the GraphPad Prism 4 software to compare mean AUC values from the designated data subsets.

## Results

### Comparison of AUC and IC_50_ values for identifying virus-neutralizing activities of anti-V3 mAbs in the U87 assay

In the first set of experiments, six of the anti-V3 mAbs (2191, 2219, 2557, 2558, 3074, and 3869) were tested against a panel of 57 psVs prepared by Monogram Biosciences, Inc. to express Env populations from patients' plasma viruses when infection was due to HIV-1 subtypes A, B, C, or D; anti-V3 mAb 447 was tested against a subset of 26 psVs from this same panel. The panel of 57 psVs was chosen at Monogram to represent Envs from different subtypes, from subjects in different geographic areas, infected by different routes and at different stages of infection, i.e., from acutely-infected and chronically-infected subjects with different profiles of disease progression (rapid progressors and long-term non-progressors). In addition to the 57 psVs, four psVs prepared from single cloned Envs of SF162, JR-CSF, NL4.3, and aMLV were tested as positive and negative controls. The target cell line used in this set of experiments was CD4+CCR5+CXCR4+ U87.

**Figure 1:**
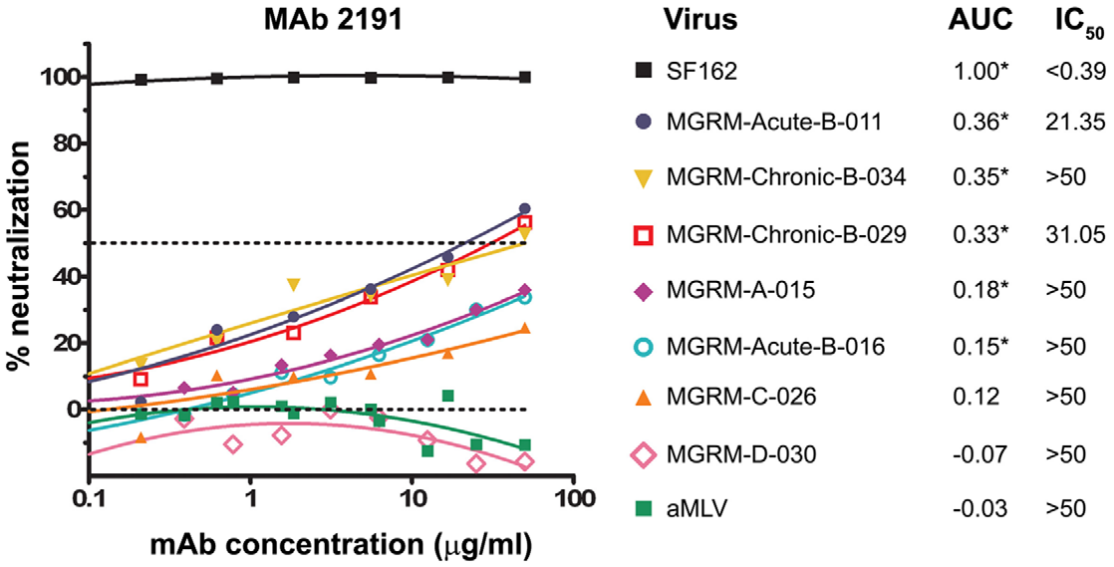
The neutralization of nine HIV-1 pseudoviruses by mAb 2191 using U87 as target cells. The neutralization curves of anti-V3 mAb 2191 against nine selected HIV-1 pseudoviruses are shown with their corresponding AUC and IC_50_ values. Fifty percent neutralization is denoted by the dashed line. Significant neutralization at the confidence level of p<0.001 (denoted with * after AUC values) was determined statistically based on comparison with the AUC values of the negative controls together with the slopes of the titration curves as described in Materials and Methods. For viruses coming from patients where the date of infection is known, viruses are denoted as coming from acutely- or chronically-infected patients, and the clade of the virus is denoted by the capital letter in its name (A, B, C or D).

To determine the neutralization in each mAb/psV combination, both the AUC and IC_50_ values were calculated from each neutralization titration curve. Figure 1 shows neutralization curves for mAb 2191 against nine representative psVs, including the negative psV control aMLV and the positive control SF162 psV; the corresponding AUC and IC_50_ values are also shown. Based on the statistical analyses of the AUC values described in the Materials and Methods, the neutralization curves for mAb 2191 against psVs SF162, Acute-B-011, Chronic-B-034, Chronic-B-029, A-015, and Acute-B-016 are classified as positive (i.e., significant neutralization, p<0.001), while the neutralization curves against psVs C-026, D-030, and aMLV are negative (i.e., neutralization is not significant).

It is noteworthy that neutralization by mAb 2191 of psVs Chronic B-034, A-015, and Acute-B-016 is considered statistically significant by the AUC analysis despite the fact that 50% neutralization was not achieved with the highest mAb concentration (50 µg/ml). For example, 50 µg/ml of mAb 2191 neutralized A-015 to a level of 36%; nonetheless, the neutralization curve shows a positive dose-response relationship and was clearly distinguishable from the data generated with mAb 2191 vs. the negative control psV, aMLV, and from the data generated vs. psV D-030 which essentially overlay those of the aMLV negative control. Thus, while the neutralizing activity of mAb 2191 against these three psVs reached a maximum of only 36% at 50 µg/ml, significant neutralizing activity was identified by statistical analysis (p<0.001).

**Figure 2:**
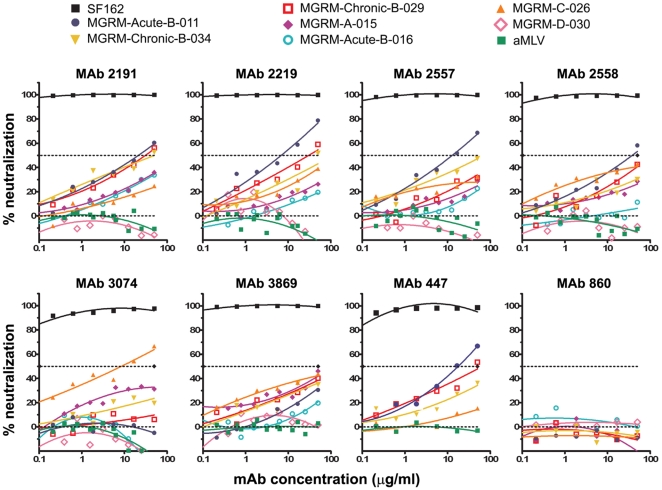
Neutralization curves of anti-V3 mAbs against representative HIV-1 psVs tested using the U87 target cell. Seven anti-V3 mAbs (2191, 2219, 2557, 2558, 3074, 3869, and 447) were tested for neutralization against HIV-1 pseudoviruses using the U87 cell line as target cells. MAb 860, specific for parvovirus, and a pseudovirus expressing the aMLV Env were used as negative controls in this assay. The titration curves from eight selected psVs and the aMLV psV control are shown. Curve fitting was performed using the polynomial regression (quadratic) model described in Materials and Methods. Fifty percent neutralization is denoted by the dashed line. Virus nomenclature is denoted as described in Figure 1.

Neutralization plots are shown in Figure 2 for seven anti-V3 mAbs and one control mAb, 860, against seven representative psVs carrying Envs from different HIV-1 subtypes, a positive control psV (SF162), and a negative psV control (aMLV). Dose-dependent neutralization was observed against a significant proportion of the psVs with each anti-V3 mAb; none of the psVs were neutralized by the control anti-parvovirus mAb 860. One hundred percent neutralization was observed with all seven anti-V3 mAbs against the positive control SF162 psV. While several mAb/psV combinations achieved >50% neutralization at the highest mAb concentration tested (50 µg/ml), for many combinations, 50% neutralization was not attained at this concentration even though dose-response relationships were displayed by the neutralization curves which were clearly above background (see below).

**Figure 3:**
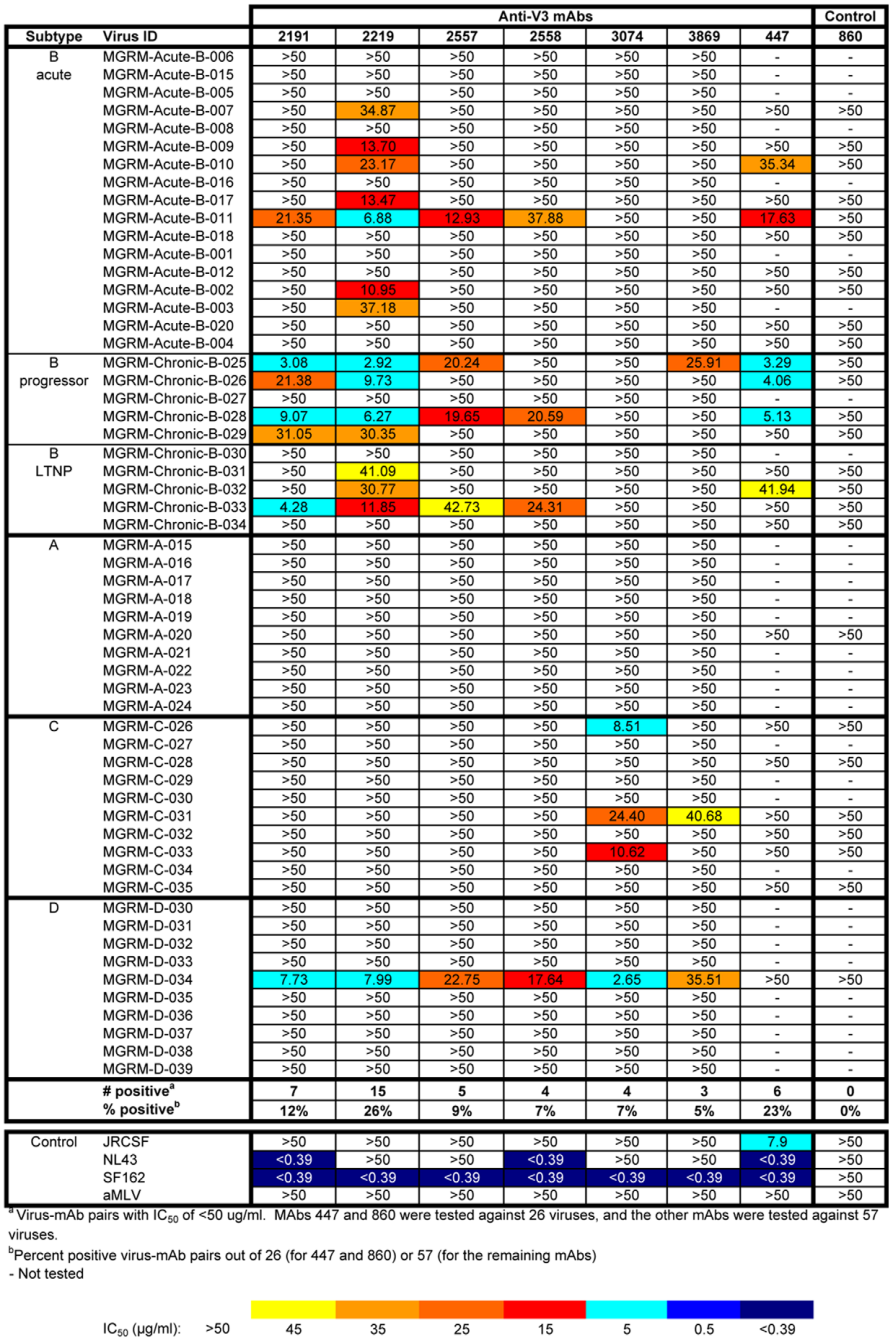
The IC_50_ values of anti-V3 mAbs against 57 HIV-1 pseudoviruses tested using the U87 target cell line. The IC_50_ values were estimated from the titration curves of all mAb/psV combinations and are highlighted according to the color-coded scale. Pseudoviruses expressing Envs of JRCSF, NL3.4, and SF162 were tested as positive controls, whereas the irrelevant anti-parvovirus mAb 860 and aMLV Env-expressing psV were used as negative controls. When 50% neutralization was not achieved at the highest mAb concentration tested (50 µg/ml), the IC_50_ values are shown as >50.

**Figure 4:**
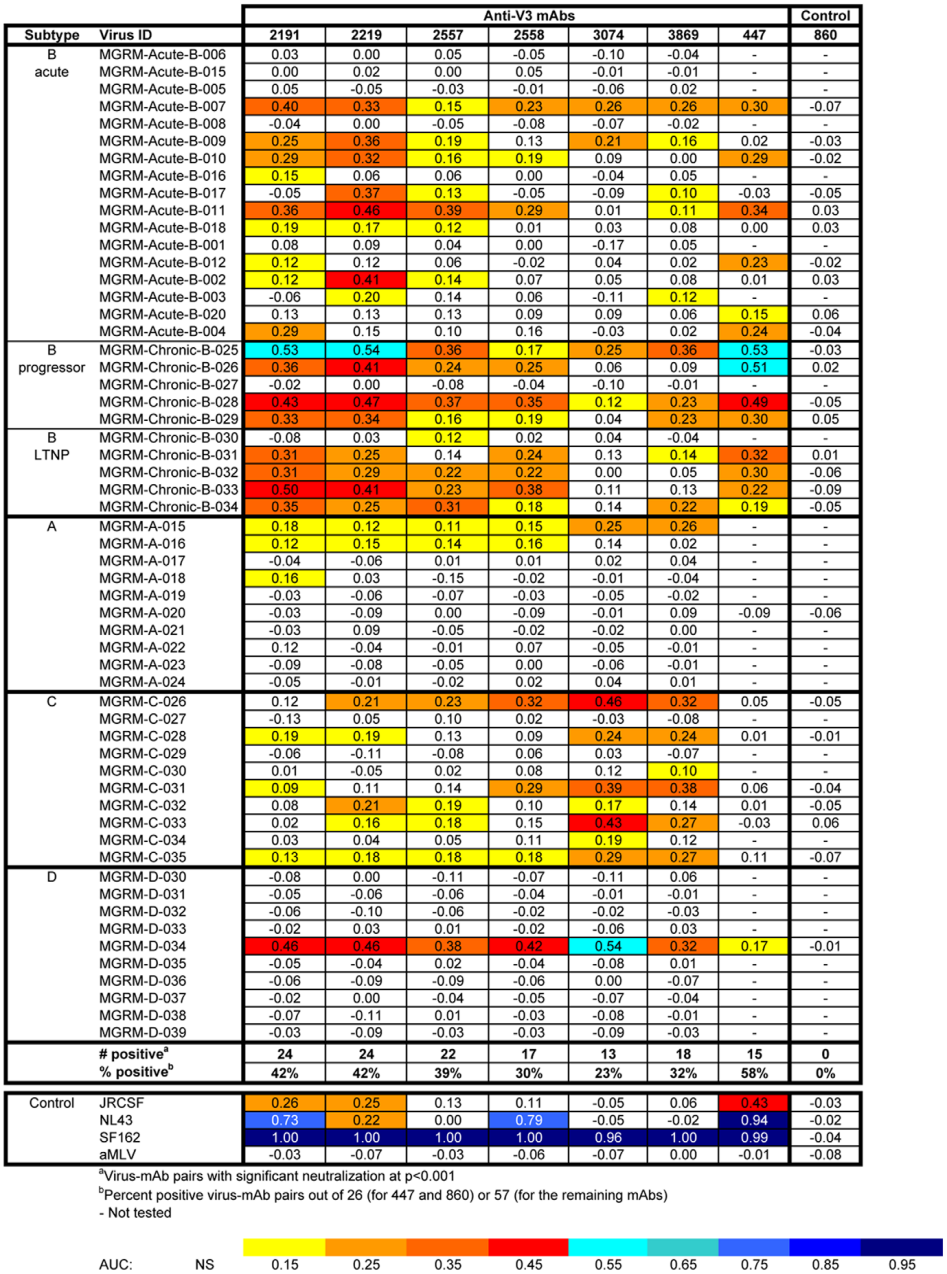
The AUC values of anti-V3 mAbs against 57 HIV-1 pseudoviruses tested using the U87 target cell line. AUC values were estimated from the titration curves as described in the Materials and Methods section. Statistically significant neutralization at p<0.001 is color-coded according to the designated scale.

When IC_50_ values were calculated for all mAb/psV combinations tested in the U87 assay system (Figure 3), 18 of the 57 (32%) psVs were neutralized by one or more anti-V3 mAbs at ≤50 µg/ml, with IC_50_ values ranging from 2.65 to 42.73 µg/ml. Statistical analysis was then performed using the AUC method described above. Figure 4 shows the same neutralization matrix as that shown in Figure 3 but with AUC values. The AUC values of all mAb/psV combinations tested, including the negative and positive controls, ranged from −0.17 to 1.00. Significant neutralization was observed against 33 out of 57 (58%) psVs by ≥1 anti-V3 mAbs. Note that significant neutralization was not determined by a particular cut-off value, but rather by the Wald test and the slope criteria described in the Materials and Methods section. Therefore, neutralization curves with the same AUC values may not be equally significant due to differences in the slopes and the variance of the fitted curves.

Thus, use of the AUC-based statistical analysis resulted in the detection of nearly twice as much neutralizing activity in this panel as did analysis by IC_50_. The difference was primarily due to low level neutralizing activity. For example, no neutralization was detected against clade A psV MGRM-A-015 by any of the seven mAbs when measured by IC_50_ (Figure 3), but 6 of 7 mAbs showed significant neutralization of this psV by the AUC analysis (p<0.001; Figure 4).

### Neutralizing activities of anti-V3 mAbs against multiple HIV-1 subtypes are detected in the U87 assay

The neutralizing activities of the seven mAbs were observed across psVs carrying the Envs of the four subtypes tested in the U87 assay. Twenty-one of the 27 (78%) psVs with subtype B Envs from acute and chronic infections and 8 of the 10 (80%) psVs with subtype C Envs were neutralized by at least one anti-V3 mAb (Figure 4). In contrast, subtypes D and A psVs were less sensitive to neutralization, with only one (10%) and three (30%) of these psVs sensitive to the anti-V3 mAbs, respectively.

The median IC_50_ value for anti-V3/psV pairs with an IC_50_<50 µg/ml in this set of experiments was 18.65 µg/ml (Figure 3), and the median AUC value for anti-V3/psV pairs with significant neutralization was 0.24. This AUC value was essentially identical to the median AUC for the anti-V3 mAbs that neutralized JR-CSF (median AUC = 0.26). As expected, this value is lower than that obtained for psVs SF162 and NL43 (median AUCs = 1.00 and 0.76, respectively); these psVs are known to be highly sensitive to neutralization. Notably, the median AUC values of mAbs that significantly neutralized subtype B and subtype C psVs (0.25 and 0.21, respectively) were comparable, indicating that the anti-V3 mAbs neutralize subtypes B and C viruses with similar potencies. It is also noteworthy that none of the mAbs tested were derived from subtype C-infected patients, yet they effectively neutralized 8 of the 10 clade C psVs.

### Neutralizing activities of anti-V3 mAbs against multiple HIV-1 subtypes are also detected in the TZM.bl assay

In a second set of experiments, the same anti-V3 mAbs were tested for their ability to block infection of TZM.bl cells by 41 psVs carrying single cloned HIV-1 Envs, including ten Tier 1 Envs (subtypes A, AG, B and C), 12 Tier 2 subtype B Envs, and 12 Tier 2 subtype C Envs. The Tier 1 and Tier 2 Envs have been selected for use in standard psV panels of neutralization assays. The Tier 2 Envs were cloned out of primary HIV-1 isolates from acute and early infection and were moderately resistant to polyclonal and monoclonal Ab reagents, while the Tier 1 Envs were highly sensitive to neutralization [50], [51]. In addition, psVs were tested carrying cloned Envs from seven clade B viruses isolated from chronically-infected individuals. As negative controls, four mAbs specific for anthrax or parvovirus antigens (860, 1418, 3685, and 3706) were used to establish background levels of neutralization.

**Figure 5:**
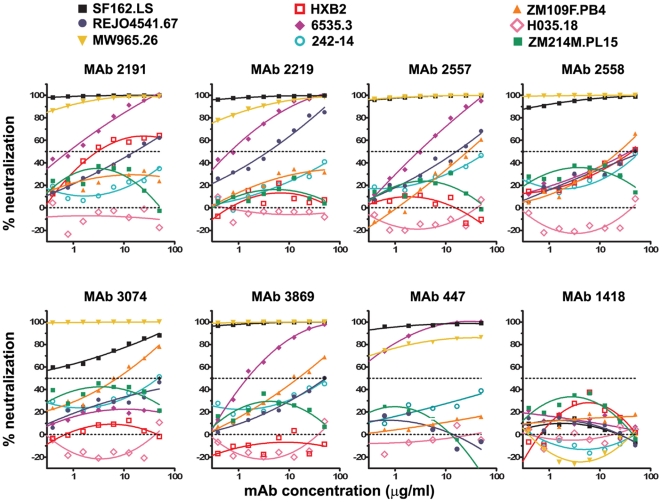
Neutralization curves of anti-V3 mAbs against representative HIV-1 psVs tested using the TZM.bl target cell line. Seven anti-V3 mAbs were tested against HIV-1 psVs in the TZM.bl experiment. The titration curves observed against nine selected viruses are shown. The background neutralization observed with the irrelevant control mAb 860 is also shown for comparison. Curve fitting was performed using the polynomial regression (quadratic) model described in Materials and Methods. Fifty percent neutralization is denoted by the dashed line.

The curves depicting the neutralizing activities of the seven anti-V3 mAbs and the negative control mAb 1418 against nine of the 41 psVs are shown in Figure 5. Generally higher levels of neutralization were detected in the TZM.bl assay than in the U87 assay described above. For example, in the TZM.bl assay, but not the U87 assay, neutralization levels >80% were frequently observed. However, the background values from the negative control mAbs were also higher in the TZM.bl assay than those generated in the U87-based experiments (Figure 5 vs. Figure 2). Comparably high background levels of neutralization were also observed with the other three negative control mAbs tested in the TZM.bl assay (data not shown). One should note, however, that the high background levels observed in the TZM.bl assay might be attributable to the particular psVs tested in the panel and not only to the assay or target cells. For instance, background neutralization of >20% was consistently observed with psVs ZM214M.PL15 and HXB2, but not with psVs MW965.26, 242-14, and H035.18 (Figure 5).

**Figure 6:**
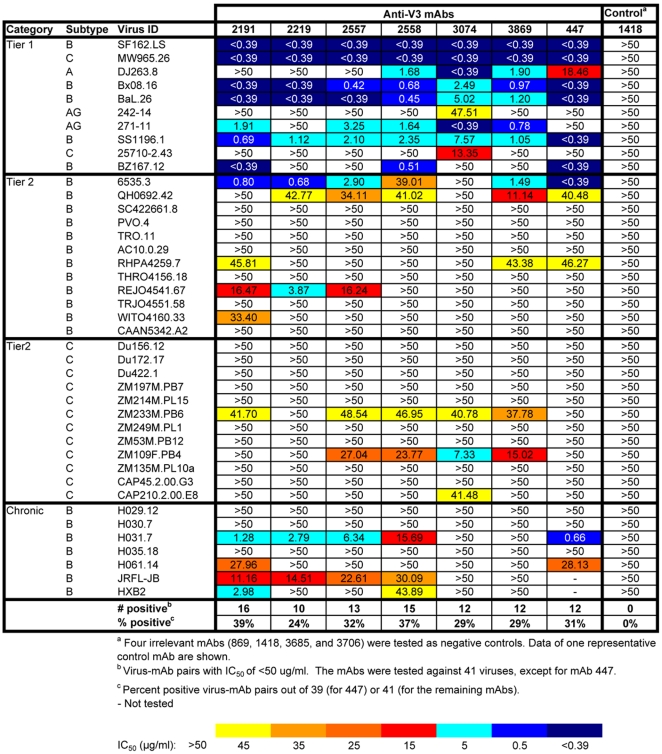
The IC_50_ values of anti-V3 mAbs against 41 HIV-1 pseudoviruses tested using the U87 target cell line. The IC_50_ values were estimated from the titration curves of all mAb/psV combinations and are shown with the color-coded scale. When 50% neutralization was not achieved at the highest mAb concentration tested (50 µg/ml), the IC_50_ values are shown as >50. Four different irrelevant mAbs (860, 1418, 3685, and 3706) were used as negative controls in this experiment with comparable results, but only the mAb 860 data are shown.

**Figure 7:**
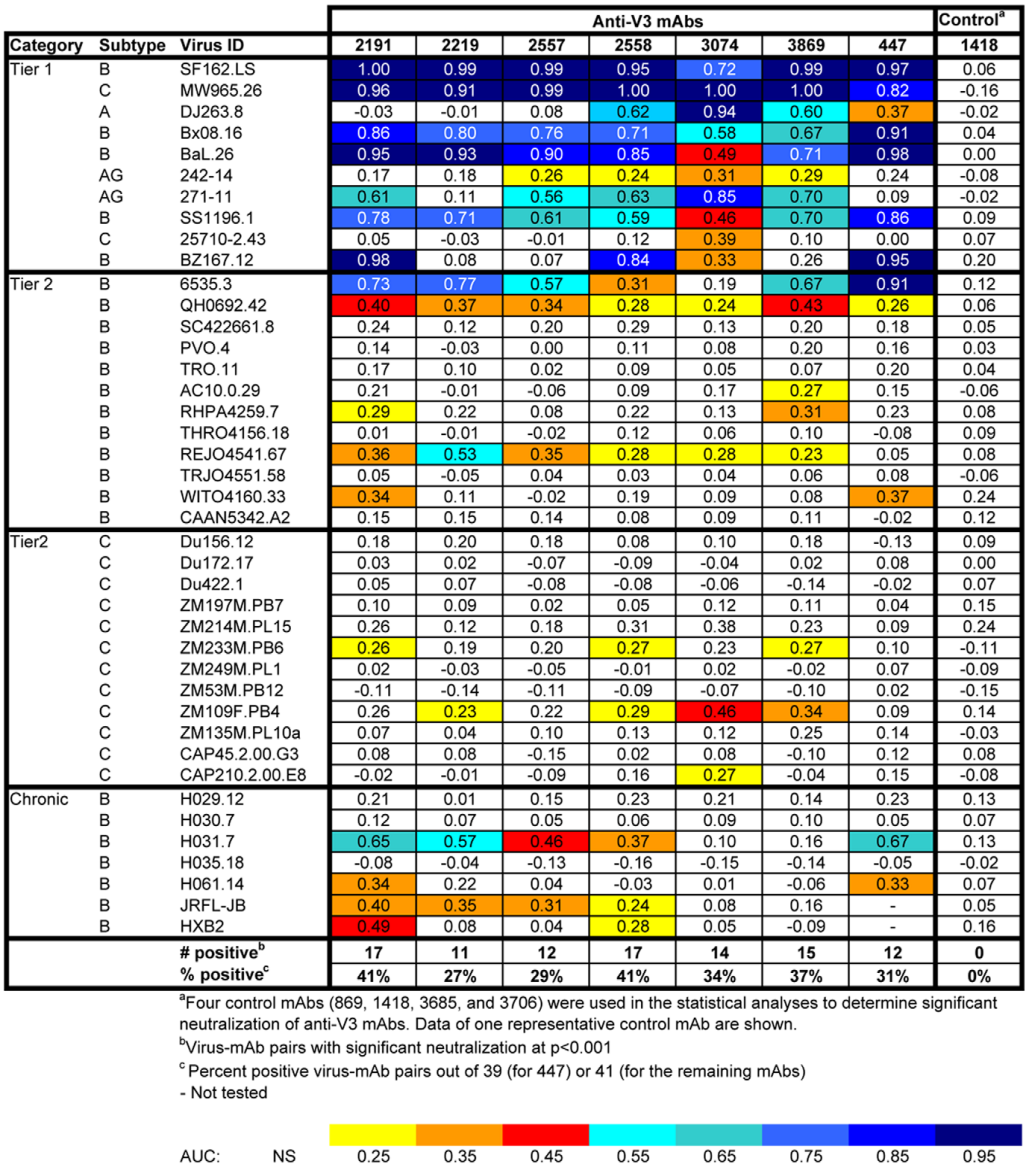
The AUC values of anti-V3 mAbs against 41 HIV-1 pseudoviruses tested using the TZM.bl target cell line. The AUC values were calculated from the titration curves as described in the Materials and Methods section. All mAb/psV pairs with statistically significant neutralization at p<0.001 are color-coded according to the designated scale. The irrelevant anti-parvovirus mAb 1418 was used together with three other control mAbs (data not shown) as negative controls.

When 50% neutralization was used as a cut-off for positive neutralization with the TZM.bl experimental data, 22 of 41 (54%) psVs were neutralized, with IC_50_ values for neutralizing mAbs ranging from <0.39 to 48.54 µg/ml (Figure 6). The median IC_50_ value for all mAb/psV combinations with IC_50_ of <50 µg/ml was 1.91 µg/ml. When calculated for the 31 Tier 2 and chronic psVs (i.e., excluding the data from Tier 1 psVs), the median IC_50_ was 23.77 µg/ml. When assessed by AUC (Figure 7), the results were similar: 23 of 41 (56%) psVs were neutralized, with significant AUC values ranging from 0.23 to 1.00. No significant neutralization was measured using either IC_50_ or AUC with any of the four negative control mAbs (Figures 6 and 7, and data not shown).

Using the AUC analysis, the Tier 1, Tier 2, and chronic viruses showed different patterns of neutralization by the anti-V3 mAbs in the TZM.bl assay. All 10 of 10 Tier 1 psVs with Envs from subtypes A, AG, B and C were neutralized by ≥1 anti-V3 mAbs (Figure 7). Indeed, a single anti-V3 mAb (3074) was able to neutralize all of the 10 Tier 1 psVs, while each of the remaining six anti-V3 mAbs neutralized 5 to 9 of the 10 Tier 1 psVs. The AUC values achieved against the Tier 1 psVs were high, with a median of 0.78 (range 0.24 to 1.00) for all positive mAb/psV combinations. Moreover, 20 of the 70 mAb/Tier 1 psV combinations had AUC values of 0.90 to 1.00.

One or more anti-V3 mAbs were also able to neutralize six of 12 (50%) clade B Tier 2 psVs, with a median AUC value of 0.35 (range 0.23–0.91) for all the positive mAb/psV combinations. These data parallel those shown above (Figure 4) in which anti-V3 mAbs neutralized 12 of the 17 (71%) subtype B psVs with Envs derived from acutely-infected patients tested in the U87 assay, a group of psVs that meet the criteria for Tier 2 viruses. Among the seven chronic subtype B psVs tested in the TZM.bl assay, four (57%) were also neutralized by ≥1 anti-V3 mAbs (median AUC = 0.37, range 0.24–0.67) (Figure 7). In comparison, 90% of psVs with subtype B Envs from chronically-infected progressors and long-term non-progressors tested in the U87 assay were neutralized by anti-V3 mAbs (Figure 4). The results in the two assays show qualitatively that anti-V3 mAbs can neutralize Tier 2 psVs from clade B as well as psVs derived from the viruses of chronically-infected individuals. The quantitative differences between the two assays may be attributable to the particular panel of psVs tested.

Among 12 psVs with clade C Tier 2 subtype C Envs, statistically significant neutralization was achieved against three (ZM233M.PB6, ZM109F.PB4, and CAP210.2.00E8) in the TZM.bl assay. The neutralization of ZM233M.PB6 and ZM109F.PB4 was mediated by three and four anti-V3 mAbs, respectively, while CAP201.2.00E8 was neutralized by only one (mAb 3074) (Figure 7). In contrast, 8 of 10 (80%) subtype C psVs were neutralized by these same anti-V3 mAbs in the U87 experimental set (Figure 4). Here the comparison is not as direct, since the clade C Tier 2 panel used in the TZM.bl assay was derived from individuals recently infected with subtype C viruses, whereas the Envs of clade C psVs tested in the U87 experiments came from individuals whose date of infection was not known, but were likely to be in the chronic stage of infection.

### Combined data from the U87 and TZM.bl experiments demonstrate broad neutralizing activities of anti-V3 mAbs across Envs from different subtypes and from different stages of infection

By applying the same AUC-based statistical analyses to the two independent experiments, we observed that, 56 of 98 (57%) psVs tested were neutralized significantly by ≥1 anti-V3 mAbs (Table 2). These 56 sensitive psVs expressed Envs of diverse subtypes, including subtypes A, AG, B, C and D. Even when the 10 Tier 1 psVs were excluded from the combined data, significant neutralization was detected by ≥1 anti-V3 mAbs against 46 of 88 (52%) psVs from subtypes A, B, C and D. Furthermore, 9 of 24 (37.5%) Tier 2 viruses from the clade B and C standard panels were neutralized by ≥1 anti-V3 mAbs. Hence, the ability of anti-V3 mAbs to neutralize across different subtypes is observed consistently in both U87 and TZM.bl experiments. This establishes the ability of many anti-V3 mAbs to display cross-clade neutralizing activity and demonstrates conclusively that anti-V3 Abs can be broad in their reactivity and are not exclusively type- or clade-specific.

**Table 2:** Summary of data on psV neutralization by anti V3 mAbs.

Env subtype	Category or stage of infection	No. psVs neutralized*	Total psVs tested	% neutralized	Assay
All		56	98	57%	U87 & TZM.bl
All except Tier 1		46	88	52%	U87 & TZM.bl
A, AG, B & C	Tier 1	10	10	100%	TZM.bl
B & C	Tier 2	9	24	38%	TZM.bl
B	Acute	12	17	71%	U87
B	Tier 2	6	12	50%	TZM.bl
B	Chronic (Progressor & LTNP)	9	10	90%	U87
B	Chronic	4	7	57%	TZM.bl
A	unknown	3	10	30%	U87
C	Tier 2	3	12	25%	TZM.bl
C	unknown	8	10	80%	U87
D	unknown	1	10	10%	U87

*Positive neutralization defined by p<0.001 in AUC analysis with ≥1 anti-V3 mAb(s).

Subtype B psVs with Envs from viruses derived from chronically- and acutely-infected subjects were compared for neutralization sensitivity by anti-V3 mAbs. The data from both U87 and TZM.bl experimental sets consistently show that anti-V3 mAbs were effective against significant fractions of psVs with Envs from either chronic or acute infections. In the U87 experiments, 90% (9/10) and 71% (12/17) of psVs with Envs from chronic and acute infections, respectively, were neutralized by ≥1 anti-V3 mAbs (Figure 4 and Table 2). The mAbs showed a trend toward better potency against psVs with chronic Envs (AUC = 0.12 to 0.54; median  = 0.30) than against acute Envs (AUC = 0.10 to 0.46; median  = 0.23); this difference did not reach statistical significance. In the TZM.bl experiments, psVs with subtype B Envs from both chronic (4/7 [57%]) and acute (Tier 2; 6/12 [50%]) infections were also sensitive to neutralization by anti-V3 mAbs (Figure 7 and Table 2), and there was essentially no difference in the potency of the mAbs against viruses with chronic Envs (AUC = 0.24–0.67, median  = 0.37) vs. those with acute Envs (Tier 2; AUC = 0.23–0.91, median  = 0.35).

### Different anti-V3 mAbs display unique patterns of neutralization

Each anti-V3 mAb tested was able to neutralize 28–42% of the 98 psVs tested, whereas the four anti-parvovirus and -anthrax mAbs used as negative controls did not neutralize any of the psVs. However, the data in Figures 4 and 7 clearly show that each anti-V3 mAb displays a unique pattern of psV neutralization. To illustrate these distinct patterns of neutralization, data from three mAbs, 2191, 3074, and 447, are described in detail this section.

Based on statistical analyses of the AUC data, mAb 2191 was found to neutralize the highest proportions of psVs, and this was consistently observed in both the U87 and TZM.bl assays (Figures 4 and 7). Twenty-four of 57 (42%) and 17 of 41 (41%) psVs in the respective panels were significantly neutralized by mAb 2191. Importantly, this single mAb displays neutralizing activity across multiple subtypes, including psVs carrying Envs from Tier 1, Tier 2, acute and chronic viruses from subtype B, from Tier 2 and chronic viruses from subtype C, from chronic clade A and D viruses, and from Tier 1 AG viruses. The frequency of subtype B psVs neutralized by mAb 2191 (which was derived from a clade B-infected individual) was greater than that for the non-B psVs, and the potency against the subtype B psVs was also notably stronger. This inter-subtype neutralizing activity was not unique to mAb 2191. The other five anti-V3 mAbs (2219, 2557, 2558, 3074, and 3869) were also effective against psVs expressing different Env subtypes, as they each neutralized 28–36% of the 98 psVs tested at levels that were statistically significant. These data demonstrate that a single anti-V3 mAb has the capacity to mediate neutralization against HIV-1 of diverse subtypes.

Previous studies have shown that anti-V3 mAbs derived from African donors infected with HIV-1 isolates of non-B subtypes show different patterns of psV neutralization than those derived from subtypes B-infected subjects, and that the non-B derived mAbs have a tendency to exhibit broader and more potent neutralization against non-B viruses [18]. Of the four non-B derived mAbs studied here, mAbs 3074 and 3869 exhibited remarkable neutralization patterns: these two mAbs neutralized eight of the 10 subtype C psVs in the U87 experiments (Figure 4). MAb 3074, for example, which was derived from a clade AG-infected individual, displayed considerable breadth, neutralizing 7 of 10 clade C psVs with AUC values ranging from 0.17 to 0.46 (Figure 4); when 50% neutralization was achieved, IC_50_ values ranged between 8.51 and 24.20 µg/ml (Figure 3). This mAb also neutralized two clade C Tier 2 psVs tested in the TZM.bl experiments, with AUC values of 0.27 and 0.34 (Figure 7). Using the IC_50_ criterion, three clade C Tier 2 viruses were neutralized, with IC_50_ values of 7.33 to 41.48 µg/ml (Figure 6). In contrast, the neutralization of mAb 3074 against acute, chronic, and Tier 2 subtype B psVs was more sporadic and less potent. These data provide clear evidence for the distinct and complementary specificities of neutralizing activities mediated by the individual anti-V3 mAbs and suggest that increased breadth of virus neutralization can be attained by combinations of selected anti-V3 mAbs.

In contrast to the six anti-V3 mAbs described above, the anti-V3 mAb 447, which has, until now, been considered as the prototypic anti-V3 mAb, displays more limited breadth of neutralization across subtypes. Although mAb 447 significantly neutralized 27/65 (42%) psVs in the U87 and TZM.bl experiments, it was effective mainly against psVs with subtype B and Tier 1 Envs (Figures 4 and 7). This is strikingly similar to the data published previously showing that mAb 447 neutralized 38% and 45% of various clade B psVs panels but a very small proportion of psVs from other subtypes [30], [31], The data suggest that mAb 447 tends to be a “clade B-specific” anti-V3 mAb, and is clearly distinct from the other much more broadly reactive anti-V3 mAbs tested in these studies. This finding is consistent with previously published data showing that the V3 motif critical for mAb 447 recognition is the presence of Arg^315^ (R) at the GPGR arch of the V3 crown [40], [44], [53] and that this motif is generally restricted to subtype B viruses, whereas the typical sequence at the V3 arch of the other subtypes is GPGQ.

## Discussion

By testing seven different anti-V3 mAbs against 98 psVs with either single cloned Envs or cloned Env populations in two independent laboratories, and utilizing neutralization assays with different target cells, this study has demonstrated the ability of human anti-V3 mAbs to neutralize diverse HIV-1 strains from multiple subtypes derived from patients at different stages of infection. Overall, the data show that 57% of the 98 psVs tested were sensitive to neutralization by one or more of the seven anti-V3 mAbs. Any single anti-V3 mAb was capable of neutralizing 28–42% of these psVs. Given the reactivity of these monoclonal reagents, the data suggest that polyclonal anti-V3 Ab responses will neutralize an even greater proportion of HIV-1 isolates than any single anti-V3 mAb or cocktail of anti-V3 mAbs. Indeed, this hypothesis is corroborated by recent findings showing that sera from rabbits that were primed with gp120 DNA and boosted with a V3-fusion protein exhibited a greater breadth of neutralizing activity than pools of anti-V3 mAbs [32]. The accumulated data on the breadth of the neutralizing activity of anti-V3 mAbs, from immune sera induced with a vaccine focusing the Ab response on V3 and from polyclonal serum anti-V3 Abs from infected individuals [54] suggest that V3 is one of the Env epitopes of HIV-1 that should be targeted by vaccines.

The extent of cross-reactivity displayed by the anti-V3 mAbs is not surprising in light of the structural and bioinformatics data published in recent years which show the presence of several conserved structural elements in the V3 loop. Huang et al. [43] demonstrated three regions of the V3 loop: a base which is attached to the gp120 core, a flexible stem, and a crown, consisting of ∼14 amino acids at the center of the loop which contains all epitopes recognized by anti-V3 mAbs [40], [41], [42], [44], [55], [56], [57]. One of the conserved features of the V3 crown is the presence of the GPG motif at the tip of the loop that propels the distal tip of the V3 crown to adopt a unique β-hairpin structure. MAb 447, for example, interacts with this conserved GPG turn via hydrophobic interactions, and with the main chain of the N-terminal V3 β-strand flanking the GPG turn, rendering this mAb unaffected by side-chain differences in the highly variable V3 N-terminal β-strand [40], [42], [53]. These studies also reveal that the reactivity of mAb 447 is restricted by a polar interaction with the side chain of the R residue in the GPGR motif found mainly among viruses of subtype B but infrequently in other subtypes.

From the crystal structures of V3 peptides bound by different mAbs, additional conserved elements in the V3 epitopes have been identified that provide the structural basis for anti-V3 cross-reactivity. The cross-reactivity of mAb 2219, for example, is due to its ability to recognize conserved residues on the hydrophobic face of V3, composed of residues flanking the GPG tip [41], and further studies indicate that the 2219 epitope occurs in 30% of worldwide isolates [53]. Additional data based on viral bioinformatics and modeling studies demonstrate that the variability in V3 is, in fact, restricted to a small zone on the surface of the hydrophilic face of the V3 loop β-hairpin [58]. Thus, there is considerable structural conservation of V3, which is consistent with the requirement for V3 to participate in coreceptor binding regardless of the amino acid sequence variability this region displays.

It is noteworthy that the breadth attained by individual anti-V3 mAbs assessed by IC_50_ values, i.e., 28% to 42% against 98 psVs, is not dissimilar to that of other broadly neutralizing mAbs such as b12 and 2G12 which, respectively, neutralized 35% and 32% of 162 viruses tested in the same U87 assay and assessed by the same criterion [15]. It is also comparable with the breadth of neutralization recently reported for mAb HGN194, which recognizes a conserved epitope in the V3 crown [59]. The anti-MPER mAbs 2F5 and 4E10 show broader activity (60% and 98%, respectively), as do the newly isolated mAbs PG9 (79%) and PG16 (73%) that recognize quaternary neutralizing epitopes composed of portions of the V2 and V3 loops on HIV-1 virions. As noted above, however, the polyclonal response to a neutralizing domain such as V3 or the CD4 binding site may have much greater breadth than that displayed by any single mAb, or indeed, by a cocktail of mAbs.

The issue of the concentration of individual mAbs and polyclonal Abs needed for protection *in vivo* has received much attention, and the consensus has undergone vast changes as new data have emerged. Whereas early passive immunization experiments with mAbs suggested that extremely high levels of serum Ab concentrations were needed for protection [60], [61], recent data from a SHIV/macaque model suggest that as little as 30–60 µg/ml of an effective mAb may be sufficient to protect against a low dose challenge, comparable to that occurring in nature [62]. Thus, the levels of potency offered by the various broadly neutralizing anti-gp41 and gp120 mAbs, including those specific for V3, may well offer the requisite protection given their median *in vitro* IC_50_ values of <30 µg/ml (see Results and [15]).

In addition to providing data documenting the breadth and potency of anti-V3 mAbs, this study demonstrates the utility of a new method for objective statistical analysis of neutralization data. In the past, low levels of Ab-mediated neutralization have been essentially overlooked, as neutralizing activities have conventionally been presented as the titers or Ab concentrations required to reach various arbitrary neutralization levels; indeed, inhibitory Ab concentrations for 40–100% neutralization have been used in various HIV studies. If background levels for negative controls approach 50% neutralization, as in the case of the TZM.bl assay (see the Figure 5 panel showing data from assays with the negative control mAb 1418), then using IC_50_ values approximate statistical significance and little neutralizing activity is missed. This agrees with a recent report demonstrating a high correlation of IC_50_ titers with partial AUC values (defined as the areas of the titration curves measured between 20 and 100% neutralization) for a large panel of psVs tested using TZM.bl target cells [63]. Thus, in the data from the TZM.bl assay, the IC_50_ values and AUC analysis show, respectively, 54% and 56% of psVs neutralized by one or more anti-V3 mAbs. However, if, as in the case of the U87 assay, background levels rarely exceed 20% neutralization (see the Figure 2 panel showing data from assays with the negative control mAb 860), then using IC_50_ values censor all of the data between background and 50%, resulting in an underestimate of neutralizing activity. In this latter case, the use of AUC more comprehensively assesses neutralizing activity. In the data from the U87 assay, the proportions of psVs neutralized are 32% and 58% when analyzed by IC_50_ and AUC, respectively. Thus, one advantage of using the AUC method is its ability to detect low levels of neutralizing activity that might otherwise be missed and might be biologically relevant.

There are several additional advantages to the AUC analysis of neutralization data. 1) It allows statistically-based assessment of all neutralization data, with no preconceived cut-off points. This method takes into account the entire data from each titration curve, the background neutralization for a given assay, and the slope of the curve. It then utilizes a statistical test to determine significant activity. 2) The AUC summarizes neutralization responses across a range of Ab concentrations without requiring assumptions about the sigmoidal shape of the titration curve. Indeed, the majority of neutralization curves are not sigmoidal (Figures 1, 2, and 5). 3) AUC does not rely on a single point in various neutralization curves which may or may not be in the linear portion of each of the curves. 4) All data are used, and none are censored, Thus, Abs that do not attain the selected level of neutralization at the highest concentration tested (e.g. 50 µg/ml) are not “censored”, i.e., listed as “<50”. And, 5) a uniform, AUC-based statistical test can be applied for analysis and comparison of multiple data sets from independent experiments. This approach may therefore prove useful for analyses of neutralization data from preclinical and clinical trials that evaluate incremental improvements in the designs of candidate HIV/AIDS vaccines as they are being optimized.
